# Editorial Notes

**Published:** 1904-08

**Authors:** 


### EDITORIAL NOTES.

Dr. W. H. Whitslar who has been the efficient secretary of the Dental Department of Western Reserve University has severed his connection with that school.

---

As we go to press we learn with deep regret that Dr. Geo. H. Chance, one of the leading dentists of Portland, Oregon, and the author of Sunshine Thoughts for Gloomy Hours is not expected to live.

---

At a special meeting of the Faculties Association held at St. Louis, July 18th, it was decided to return to a three years' course in dentistry, the course to be thirty teaching weeks in each year.

---

Dr. A. W. Harlan who has practiced for so many years in Chicago has removed to New York City.

---

The faculty of the Pittsburg Dental College has undergone a considerable change during the past year.

Dr. H. E. Freisell is the new dean, and with him are associated some of the best men in that section.

